# Disturbance Alters the Phylogenetic Composition and Structure of Plant Communities in an Old Field System

**DOI:** 10.1371/journal.pone.0007071

**Published:** 2009-09-18

**Authors:** Russell Dinnage

**Affiliations:** Department of Ecology and Evolutionary Biology, University of Toronto, Toronto, Ontario, Canada; Centre National de la Recherche Scientifique, France

## Abstract

The changes in phylogenetic composition and structure of communities during succession following disturbance can give us insights into the forces that are shaping communities over time. In abandoned agricultural fields, community composition changes rapidly when a field is plowed, and is thought to reflect a relaxation of competition due to the elimination of dominant species which take time to re-establish. Competition can drive phylogenetic overdispersion, due to phylogenetic conservation of ‘niche’ traits that allow species to partition resources. Therefore, undisturbed old field communities should exhibit higher phylogenetic dispersion than recently disturbed systems, which should be relatively ‘clustered’ with respect to phylogenetic relationships. Several measures of phylogenetic structure between plant communities were measured in recently plowed areas and nearby ‘undisturbed’ sites. There was no difference in the absolute values of these measures between disturbed and ‘undisturbed’ sites. However, there was a difference in the ‘expected’ phylogenetic structure between habitats, leading to significantly lower than expected phylogenetic diversity in disturbed plots, and no difference from random expectation in ‘undisturbed’ plots. This suggests that plant species characteristic of each habitat are fairly evenly distributed on the shared species pool phylogeny, but that once the initial sorting of species into the two habitat types has occurred, the processes operating on them affect each habitat differently. These results were consistent with an analysis of correlation between phylogenetic distance and co-occurrence indices of species pairs in the two habitat types. This study supports the notion that disturbed plots are more clustered than expected, rather than ‘undisturbed’ plots being more overdispersed, suggesting that disturbed plant communities are being more strongly influenced by environmental filtering of conserved niche traits.

## Introduction

After a disturbance which removes vegetation, plant communities are reset. From bare ground new communities sprout up and change over time as the forces of seed bank dynamics, colonization, environment and interspecific interactions act upon them. Studying successional dynamics often yields insight into these processes, which is why the process of succession in plant communities has remained an important focus of research in community ecology [1], [2]. A relatively new method of analyzing community data examines how the evolutionary history (phylogeny) of species influences community structure. I hope to show that the techniques of community phylogenetics have potential for expanding our understanding of succession.

Disturbance can change the balance of forces acting on the local community. By eliminating species and thus freeing space and resources, disturbance usually will temporarily reduce the impact of interspecific competition [3], [4], [1]. As succession occurs, the original strength of competition is gradually restored. Change in the strength of competition is thought to be one of several important drivers of changes in community composition during early succession [1]. In general, this process of recurring disturbance and successional change should promote coexistence of competing plants if there is a trade-off between competitive ability and colonization efficiency or resistance to disturbance [5].

Any change in competition can also affect the phylogenetic structure of communities [6]–[9]. Competition and ‘environmental filtering’ affect the degree of similarity in the ecological roles (‘niches’) of species in communities, and phylogenetic distance can be treated as a proxy for this similarity – more closely related species being assumed to be more alike. The concept of limiting similarity [10], [11] proposes that similarity in the resource requirements and usage by consumer species limits their ability to coexist. On the other hand similar species will also share environmental tolerances, meaning that more similar species will be more likely to coexist in any given area [12]. Thus the processes of competition and environmental filtering act in opposite directions. By using phylogeny to explore such patterns of similarity it may be possible to distinguish which process played a stronger role in that community. The balance of competition and environmental filtering is expected to change during the process of succession, and should therefore produce predictable changes in phylogenetic structure.

The strength of competition should be weaker in communities where a recent disturbance has eliminated or reduced the abundance of competitive dominants. It is known that succession occurs in abandoned agricultural fields, changing communities from a bare plowed field through to the grassland, herbaceous mixed community we usually refer to as ‘old field’, and eventually to woodlands [13], [14]. Succession from bare ground to a semi-stable old-field community occurs quickly – on the order of a few years – making it an ideal system to study succession. In eastern North American old fields, the difference between communities of plants before and after a disturbance is thought to result partially from the elimination of competitive dominant species which include several common grass species (*Poa spp.*, *Bromus spp.*, etc.) and a few large herbaceous dicots such as *Solidago spp.*, and *Aster spp.*, [15], [16]. In this study I evaluate whether the phylogenetic structure of an old field herbaceous plant community changes in a predictable manner following elimination of these dominant species. I predict that sites that have been recently disturbed will be more phylogenetically ‘clustered’ than those that have not, because reduced competition following disturbance will relax limiting similarity. In addition, disturbance might select for disturbance tolerance traits which could be phylogenetically conserved. The purpose of this study is to test this prediction of competition theory.

Several other studies have attempted to quantify the effect of a disturbance on patterns of relatedness among species. Most, however, have used taxonomic measures of relatedness and have found mixed results, with some finding that disturbance increases average relatedness [17]–[19], but others finding no difference [20]. One study found that plant communities tended to have lower phylogenetic diversity in urban areas [21], but this study encompassed the entirety of Germany, and so was conducted on a very large scale. The study described here is concerned with what happens at the patch scale within a single habitat type. This allows control of differences in habitat that might be confounded with disturbance when compared across large scales. This is important because certain types of habitat may be selected for by humans when creating anthropogenic disturbance.

I distinguish two approaches to incorporating phylogenetic information: analyzing the phylogenetic *composition* and the phylogenetic *structure* of communities. Phylogenetic composition simply incorporates phylogenetic relatedness information into traditional methods of studying communities – for example: ordination approaches – which normally treat all species as independent. Phylogenetic structure, on the other hand, is a summary of the phylogenetic information contained in each community, analogous to measures of diversity in traditional analyses [22]. Phylogenetic structure is analyzed with phylogenetic diversity indices (several are reviewed in [23]). Phylogenetic structure can be divided into phylogenetic alpha diversity (within site) and phylogenetic beta diversity (between site) [24]. I only look at phylogenetic alpha diversity in this study, however, the concept of comparing phylogenetic composition is related to phylogenetic beta diversity, because site differences can be compared, however, the method of calculating distances between sites is different.

The questions addressed here are: 1) Are recently disturbed old field communities different than undisturbed communities both in species composition and *phylogenetic* composition?; 2) Is the phylogenetic structure of recently disturbed communities systematically different than that of undisturbed communities? If so, is there evidence that reduced competition after disturbance allows communities to be more phylogenetically ‘clustered’?

## Methods

### Data Collection

I was granted permission to conduct the study on the Koffler Scientific Reserve (KSR) at Joker's Hill (King City, Ontario; http://www.ksr.utoronto.ca/jh.html), a 350 hectare property containing a mix of primary forest, secondary forest and open habitats, including a large area of old field sites. Many areas of the reserve are plowed for experiments and agriculture. I chose fields that had been plowed within the last 2 seasons as examples of recently disturbed systems (R. Dinnage, personal observation). I located 19 of these recently disturbed fields that were separated from each other by at least 50 meters. Most were separated by 100 meters or more, and were spread throughout the old field habitat on the reserve. In each of these fields a 10×10 meter plot was placed haphazardly within 10 meters of the edge of the plowed area. Each of these ‘disturbed’ plots was then paired with an ‘undisturbed’ plot of the same size from just outside the plowed area.

The community composition of herbaceous forbs was measured by surveying the presence or absence of species within four 1×1 meter quadrats placed within the four quadrants of each plot. The data from the four quadrats were later combined to the plot level, with the number of quadrats in which each species was found acting as a coarse measure of frequency (0–4). The survey was conducted between August 24–31, 2007. All analyses were based on this survey data combined with a phylogeny for all species found in the samples. The raw sample data (data S1) and the phylogeny (data S2) are included as supplementary information files.

### Phylogeny generation

I created a phylogeny for all the herbaceous old field plants I surveyed by combining a backbone tree based on the APG phylogeny (http://www.mobot.org/MOBOT/research/APweb/) generated by Phylomatic (http://www.phylodiversity.net/phylomatic/phylomatic.html) with subtrees created using downloaded sequences. I made three such trees for three families that lacked resolution on the backbone tree (Asteraceae, Lamiaceae, and Brassicaceae), using all species from these families found in the samples plus several other species which had divergence estimates between them [25].

The internal transcribed regions (ITS1 and ITS2; but not 5.8S) for each species in these sub-trees plus an outgroup species (from the hypothesized sister family) were downloaded from GENBANK [26]. After downloading I aligned them using MAFFT alignment [27] with default settings, concatenated them, then analyzed them with maximum parsimony using the PHYLIP 3.47 [28] software package (dnapars program) with default settings.

Branch lengths for the backbone tree were calculated with the BLADJ program included with Phylocom 3.41 [29]. This program assigns ages to nodes that were estimated in reference [25], and then estimates the ages of remaining nodes so that they spread evenly between the dated nodes. This method loses some phylogenetic information, but is better than simply using number of nodes separating taxa as an estimate of phylogenetic distance, especially for community phylogenies which are highly incomplete.

**Figure 1:**
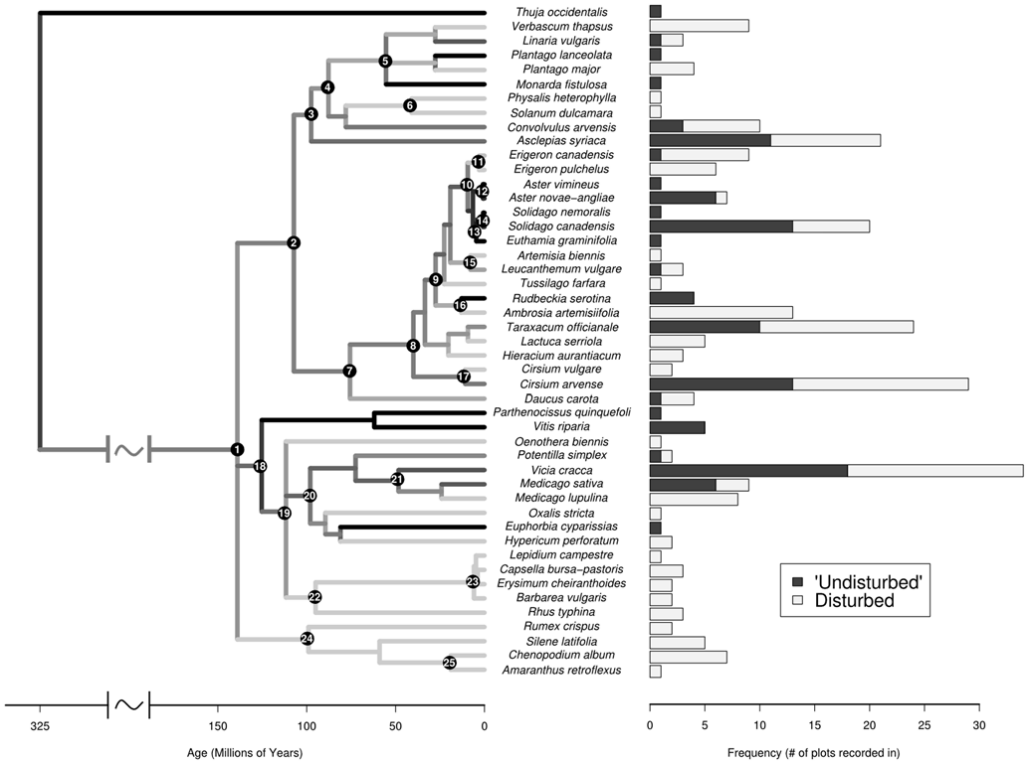
Community chronogram of old field species with their frequency in two habitat types. On the left is a community chronogram with branch lengths in millions of years. On the right is a barchart showing the relative frequencies of the species in the two different plot types. Branch colors represent the relative frequency within the plot types of the clade it connects to the tree. Darker grey is more in undisturbed plots, light grey is more in disturbed plots. Node labels: (1) Root. (2) Asterids. (3) Asterid 1. (4) Lamiales + Solanales. (5) Lamiales. (6) Solanaceae. (7) Asterid 2. (8) Asteraceae. (9) Asteroideae. (10) Astereae. (11) *Erigeron*. (12) *Aster*. (13) Goldenrods. (14) *Solidago*. (15) Anthemideae. (16) Heliantheae. (17) *Cirsium*. (18) Rosids + Vitaceae. (19) Rosids. (20) Rosid 1. (21) Fabaceae. (22) Rosid 2. (23) Brassicaceae. (24) Caryophyllales. (25) Amaranthaceae. Node names are based on the APG system of classification (http://www.mobot.org/MOBOT/research/APweb/) Note: *Solidago altissima* and *S. canadensis* have been combined into *S. canadensis* for brevity.

Branch lengths on the three subtrees were calculated from the maximum parsimony analysis and then converted into age estimates using rate smoothing in the software package r8s [30]. The estimated age of divergence of the family was assigned according to reference [25]. Several taxa that were included either in reference [25] or [31] – which estimated divergence times for groups within the Asteraceae – were included in the subtrees so that an estimated age could be assigned to the common node between them and improve the accuracy of the r8s estimate of intervening nodes. Species not occurring in the samples were then trimmed off, before I grafted these subtrees onto the backbone tree. This final tree is a nearly fully resolved (to the genus level) ultrametric tree with branch lengths in units of time (millions of year) – i.e., a community chronogram (figure 1). Measures of community phylogenetic structure were based on this phylogeny. *Thuja occidentalis* was an unusual occurrence in one plot and was removed from subsequence analysis as a extreme outlier (very large branch length and very rare). Inclusion of *Thuja* did not change the results of the analysis.

### Comparison of disturbed and undisturbed plots

I compared disturbed and undisturbed plots using several measures of community structure, both traditional and phylogenetic. All analyses were performed using R Statistical Language Environment [32].

### Species composition

I looked for a difference in species composition between disturbed and undisturbed sites using non-metric multidimensional scaling (NMDS)[33] using the metaMDS function in the *vegan* package [34]. NMDS is an ordination technique which graphically arranges communities according to their similarity in species composition. I used Bray-Curtis distance because it has been found to perform well in simulations for ecological data [35]. Plots which occur close together on the NMDS generated axes are similar in composition. For analysis I used three axes, which fit the data well and resulted in a stress of 15.1%. To test the difference between disturbed and undisturbed plots I used the envfit function from *vegan*, with plot type as a categorical factor variable. Envfit will calculate centroids for each factor level and calculate the difference between centroids. Significance of this difference is calculated using 1000 random permutations of the factor levels. If the observed difference in centroids is greater than more than 95% of the randomly permuted datasets, we can reject the null hypothesis of no difference at an alpha of 0.05.

### Phylogenetic composition

To test for differences in phylogenetic composition between disturbed and undisturbed plots I again used NMDS, but instead of species composition as the input I used a representation of the phylogenetic nodal structure of each community. To do this I used the node-as-factor function in Phylocom [29]. This function generates a matrix with a different column for every node in the input phylogeny – then for each plot sample it fills in what nodes are present in that community, weighted by their frequency. In order for a node to be considered present in a community, at least one species from the clade subtending that node must be present. The result is similar to a standard community matrix, but with columns representing phylogeny nodes instead of species. This allows the incorporation of phylogenetic information into ordination, by allowing communities that share nodes to be considered similar. In an ordinary ordination species are treated independently. For example, if two closely related species have disjunct distributions, they could cause communities to be very different on a traditional ordination. However, using a node-as-factor approach, their close relationship will cause these communities to be more closely clustered. I used three axes resulting in a stress of 11.7%. Again, envfit was used to test the difference between disturbed and undisturbed plots using 1000 permutations.

### Phylogenetic structure

I used two methods to compare phylogenetic structure amongst habitats, and take concordance between the results to be a sign of robustness.

#### Method 1

First, I calculated a phylogenetic diversity index for all the plots that could be compared amongst them. I chose two related phylogenetic diversity indices: *phylogenetic species variability* (PSV) and *phylogenetic species evenness* (PSE) [36]. PSV is calculated as the expected variation within a community for a trait that is evolving neutrally at a fixed rate (i.e. under brownian motion *sensu* Felsenstein [37]). This method was useful for comparing between habitats because it is unbiased with respect to species richness [36], [38]. This is desirable because disturbed and undisturbed habitat are likely to differ in species richness. PSE allows for the incorporation of abundance data into PSV. I used species frequency as a measure of abundance.

PSV and PSE, like all phylogenetic diversity indices, are dependent on the shape of the input phylogeny, which is a function of the species pool considered. In other words, every species pool will have a unique ‘expected’ phylogenetic diversity for plots of differing species richness. Therefore, phylogenetic diversity indices must be interpreted in terms of deviation from the expected in order to make inferences about forces acting on the plot scale (i.e. independent of the forces which created the species pool). I therefore compare phylogenetic diversity in two ways. First I compare the raw diversity values, which can tell us about the overall pattern of phylogenetic diversity amongst habitat types. I do this with a simple paired t-test for PSV and PSE. However, since raw PSV and PSE incorporate information both about the structure of the overall species pool of the habitat, in addition to structure at the plot scale, this comparison cannot distinguish the forces that are at work within each different habitat, unless we take the species pool for each habitat to be the same. Therefore, I compare how plots in each habitat deviate from their expected phylogenetic diversity based upon the species pool for that habitat by generating null distribution based upon a simple null model of community assembly.

Null distributions were generated for PSV and PSE using the *phylostruct* function in the R package ‘Picante’ [39]. This function generates null communities by randomly assembling them from the observed species pool. The null model I used randomly placed species into communities so that each species maintained its original frequency among plots. Results using other possible null models were similar. Null distributions of mean PSV and PSE were generated for 1) all plots 2) just disturbed plots, and 3) just ‘undisturbed’ plots, to which the observed mean PSV and PSE were compared.

#### Method 2

I compared the degree to which phylogenetic distance amongst species pairs was correlated with their degree of co-occurrence (as in [7]). I used the *comm.phylo.cor* function in the R package ‘picante’ [39]. This function calculates pairwise phylogenetic distances among species using an input phylogeny and then compares this to an index of species co-occurrence using correlation. I used Schoener's index of co-occurrence (‘cij’ [40]), following reference [7]. For visualizing the results I placed species pairs into ‘bins’ based on their phylogenetic distance from one another. Each bin spanned approximately 10–15 million years. I calculated the mean and standard error of the co-occurrence indices within each phylogenetic distance bin and plotted this. The raw data was used to calculate statistics for hypothesis testing.

The test was repeated for: 1) all plots, 2) just disturbed plots, and 3) just ‘undisturbed’ plot. Traditional Pearson correlation statistics were produced for each, however, the assumption of this statistic are violated. Because the comparison was conducted on pairwise measures, and each species is compared to every other species, datapoints are not independent. To control for this violation, I performed a randomization test [7]. Because I was interested in the effects of phylogeny, species were randomly shuffled amongst the tips of the phylogeny 1000 times and the Pearson correlation recalculated each time. This produced a null distribution of correlation coefficients to which the observed values could be compared. This procedure is essentially similar to a ‘Mantel’ test. I could also test if the Pearson correlation value differed significantly between habitats by calculating the difference between generated correlations for the disturbed habitat and those for the ‘undisturbed’ habitat for each 1000 iterations. This creates a null distribution for the difference between habitats in their correlation between phylogenetic distance and co-occurrence. If the actual difference is greater than 95% of the generated values, then I can conclude that it is significant at the *a* = 0.05 level. Since I expected disturbed plots to be more phylogenetically clustered, and thus have a more negative correlation between phylogenetic distance and co-occurrence I performed a one-tailed test of this hypothesis.

## Results

### Species composition

**Figure 2:**
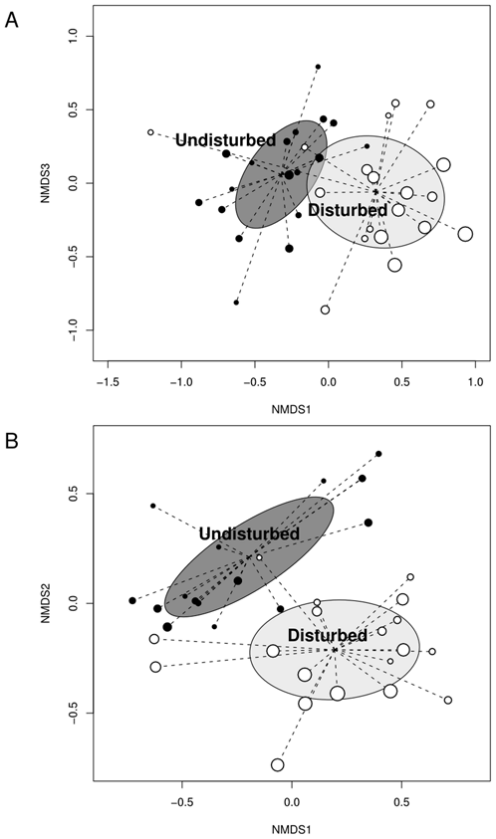
Non-metric multidimensional scaling scores for disturbed and undisturbed plots. Plot of the nonmetric multidimensional scaling scores for the two most important axes for all plots using the a) species composition data, or b) phylogenetic nodal structure data. The centroids for disturbed and undisturbed plots are labelled and linked to all points with radiating lines. Ellipses represent 1 standard deviation. Size of the points represents the relative number of species found in the plots.

Disturbed plots had nearly 70% more species than undisturbed plots (p<0.001). Nonmetric multidimensional scaling plots showed strong segregation between disturbed and undisturbed plots. The two environments separated mostly on axis 1 and axis 3 – only these axes are shown in the plot (figure 2a). The difference in centroids was significant (p<0.001).

### Phylogenetic composition

NMDS using the phylogenetic node structure of the plots showed even stronger separation between disturbed and undisturbed plots, with no overlap of the standard deviation ellipses (figure 2b). This time most of the separation occurred in the first two axes, which were plotted (figure 2b). The centroids were significantly different (p<0.001).

**Figure 3:**
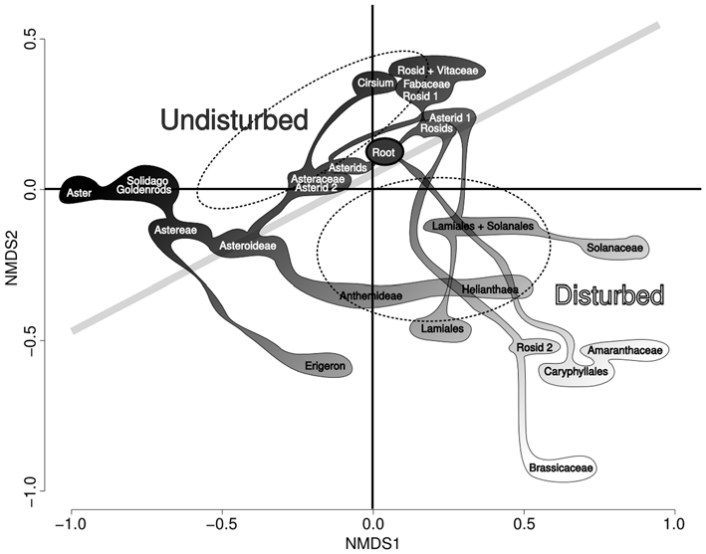
Non-metric multidimensional scaling scores for all phylogenetic nodes with reference to habitat occurrence tendency. Ordination plot for the phylogenetic ordination shown in figure 2b but with plot points removed and phylogenetic node vectors displayed. For clarity, only important or non-redundant nodes are displayed. Filled bubbles are a rough representation of the branching structure of the nodes from one another. Dark filling represents more association with undisturbed plots whereas lighter filling represents more association with disturbed plots. Dotted ellipses represent the centroids and 1 SD area as a reference for where disturbed and undisturbed plots fall on the diagram. Nodes which are referred to can be seen on figure 1.

Most of the major clades have a relatively even representation in both habitat types. This can be seen in figure 3, as the major division in the tree used here (rosids, asterids, Asteraceae) fall along the dividing line between disturbed and undisturbed habitat. Within these major groups, each smaller clade seems to dominate in one habitat or the other. On the other hand, it is very rare that two species which are each others' closest relative on the tree are found in different habitats. Therefore, habitat preference seems to be phylogenetically conserved at an intermediate phylogenetic scale.

### Phylogenetic structure

**Figure 4:**
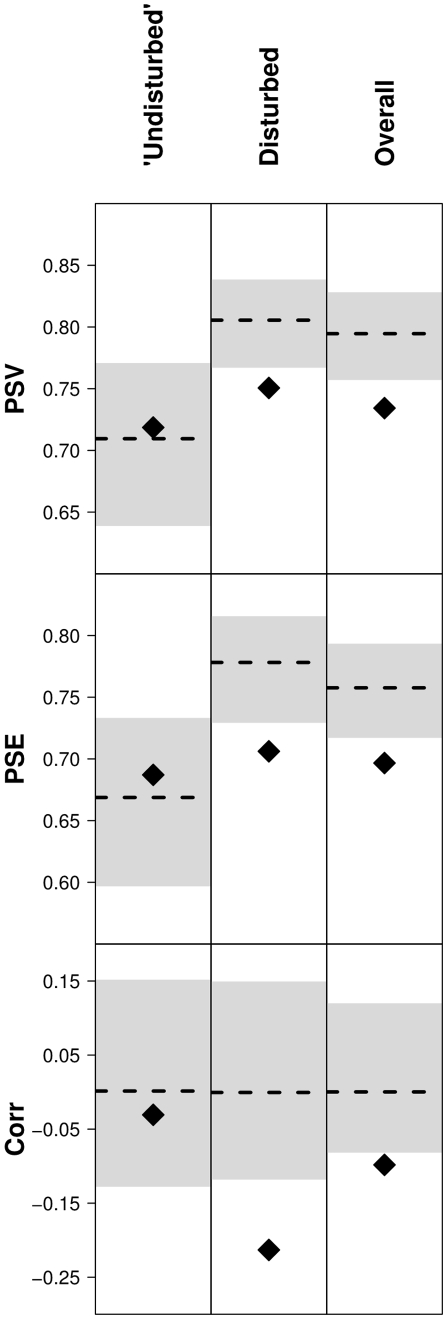
Phylogenetic structure values for disturbed and undisturbed plots, and overall. Plot showing the mean deviation in the phylogenetic structure measures for each of the plot types and overall. Diamonds represent the observed mean. Solid lines represent the expected mean based on the null model. The grey boxes surrounding the line are the 95% confidence interval from the null model.

**Figure 5:**
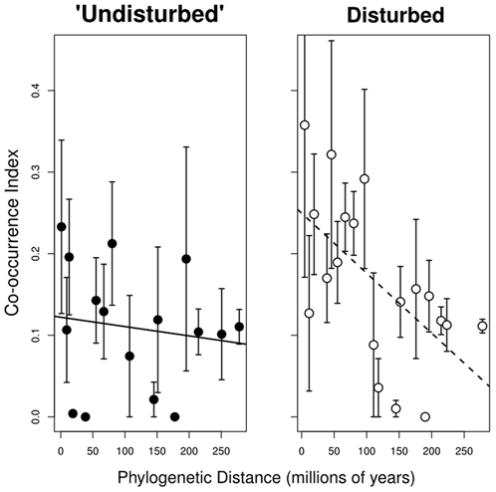
Relationship between co-occurrence and phylogenetic relatedness for disturbed and undisturbed plots. Plots showing the relationship between phylogenetic distance of species and their degree of co-occurrence for disturbed and undisturbed plots. Co-occurrence index is Schoener's index [40]. Line of best fit is included for illustrative purposes, and is based on least-squares.

There was no significant difference in PSV (t = −0.67, df = 18, p = 0.51) or PSE (t = −0.49, df = 18, p = 0.63) between habitats, though they were both slightly lower in the ‘undisturbed’ habitat (figure 4). However, habitats differed in how they deviated from expected PSV and PSE. When looking at plots across both habitats, on average both PSV and PSE were significantly lower than expected by chance under a null model of community assembly (PSV: p<0.001; PSE: p<0.001; figure 4). When considered separately, however, disturbed plot were on average significantly phylogenetically clustered – PSV and PSE were lower than expected (PSV: p = 0.008; PSE: p<0.001; figure 4). On the other hand, ‘undisturbed’ plots had a random phylogenetic structure, with no difference between observed PSV/PSE and expected based on the null mode (PSV: p = 0.59; PSE: p = 0.68; figure 4). This result was corroborated by a significant negative correlation between co-occurrence and phylogenetic distance between species in the disturbed plots (corr = −0.21, p = 0.003; figure 5), but no significant correlation in the ‘undisturbed’ plots (corr = −0.03, p = 0.32; figure 5). The difference in this correlation between habitats was also significant (p = 0.017).

## Discussion

Community composition differed between disturbed and undisturbed plots. Disturbed plots had higher species richness, consistent with previous results in old field systems [e.g 15], [16]. Nonmetric multidimensional scaling confirmed that the two habitat types differed in their species composition. This difference was exaggerated in the analysis incorporating the phylogenetic node structure of the plots. This means that disturbed plots are quite phylogenetically distinct from undisturbed plots, with each type of plot represented by different terminal clades. This can be seen visually in figure 1 and 3.

Recently disturbed plots displayed larger variation in phylogenetic ordination scores than undisturbed plots. This is likely because most species and clades that are dominant in undisturbed habitats are also found to a lesser degree in disturbed plots. Several factors are likely responsible for this, including the fact that many are perennial plants which can resprout from rhizomes after the plots were plowed. In addition there is probably a large propagule pressure from old field dominants entering into the disturbed plots. On the other hand, very few species or clades characteristic of disturbed habitats were also found in undisturbed habitats. This may be because most are annual weeds which cannot easily establish under the conditions of high density and shading in the undisturbed plots, because they are adapted to open habitats.

Overall, I found that when all plots were considered together, they were on average significantly clustered phylogenetically, when compared to a null model. This was true both for PSV, and PSE when compared to a null model of species assembly. It was also true when pairwise co-occurrence values were correlated with phylogenetic distance. This is consistent with many other studies of phylogenetic structure in plant communities, most of which have found phylogenetic clustering when structure was found [reviewed by 41], [42]. However, simulation studies have found that these tests can be liberal under several circumstances [43], including cases of spatial autocorrelation due to limited dispersal, and phylogenetic structure in the experiment-wide abundances. Abundance Phylogenetic Deviation index (APD [43]) measures the clustering of species abundance on the phylogeny by comparing the mean phylogenetic distance of species in the experiment, with the abundance-weighted mean phylogenetic distance. Positive values suggest clustering of abundances, whereas negative values suggest overdispersion of abundances. In this study the APD value for undisturbed plots was 0.08 and for disturbed plots it was 0.10 – slight clustering. This could have made the overall test liberal, however, it is the comparison among habitat types which is the important result. Since the APD values are close, there is little reason to suspect that the test for the disturbed sites is more liberal than that for the undisturbed sites, and so there should not be a higher probability of finding significant results in the disturbed plots.

Despite no difference between disturbed and undisturbed habitat types in phylogenetic diversity indices, there was a difference in how they deviated from their expected phylogenetic diversity, based on null models of community assembly (figure 4). Disturbed plots were significantly more ‘clustered’ than expected under a null model of community assembly, whereas undisturbed plots did not deviate from random expectation. This could only have come about if the ‘expected’ phylogenetic structure of each habitat differed.

In this study, I found that the available species pool for disturbed and undisturbed habitat differed, and that the phylogenetic diversity of each pool also differs. The average phylogenetic diversity expected under a null model of community assembly is reflective of the underlying phylogenetic structure of the species pool. Disturbed plots had a higher expected phylogenetic diversity, suggesting that at a regional scale disturbed areas contain lineages that are less related than in undisturbed areas. This is contrary to my expectation, as it is usually thought that disturbance should select closely related species. One possible explanation is that the undisturbed communities actually constitute a harsher environment for species due to their high level of competition, and that there may be a suite of traits that make species suited to this environment which are phylogenetically conserved. On the other hand, since I essentially only have a single sample for each habitat's pool of species, this difference may be due to chance alone. It is impossible to assess the generality of this pattern because most studies which have found higher clustering in disturbed habitats have failed to distinguish between regional species pool differences and more local plot level differences [18], [19], [21].

The results at the plot-level within each habitat type were different. Disturbed plots were more phylogenetically clustered than expected by chance, so that individual plots had, on average, lower phylogenetic diversity than their regional habitat pool. In undisturbed habitat, phylogenetic diversity in individual plots did not differ significantly from the regional habitat pool. A weaker competitive environment in recently disturbed plots could lead to more phylogenetic clustering than expected in several ways. If the environmental tolerances of species are phylogenetically conserved, then differences in the environment could act as a filter, and closely related species will be more likely to coexist. A heavy disturbance such as plowing could create a harsh or unique environment that selects for species that can tolerate these conditions [17], [44].

Stripping away dominant vegetation could lay bare environmental variation which was masked before. This could happen if the competitive stresses of the environment are strong enough that they become more important than anything else, and so in a sense, homogenize the environment. If so, such a competitive environment will act as an initial filter, reducing the species pool, but thereafter species are distributed randomly with respect to phylogeny. This could also happen if traits relating to competition are less phylogenetically conserved than those involved with dealing with abiotic stresses which may be more prevalent in recently disturbed environments.

Another possible explanation is that both environments are experiencing forces that promote phylogenetic clustering, but in the undisturbed environment there are also strong counteracting forces promoting phylogenetic overdispersion. This would happen if ecological traits related to niche partitioning were phylogenetically conserved, and that the stronger competition in undisturbed plots led to stronger niche differentiation. Opposing processes that counteract each other's effect on phylogenetic structure has been demonstrated before. One study found that when environmental factors were statistically removed from sunfish communities, phylogenetic overdispersion was revealed [38]. An argument against this possibility is that phylogenetic conservation of niches has been difficult to demonstrate in plants. For example a study of meadow communities showed that phylogenetic distance was not correlated with niche seperation along several axes of soil conditions [45]. Another study came to the conclusion that the intensity of competition between plant species pairs was only weakly correlated with phylogenetic distance in a meta-analysis of pot experiments, and only for certain taxa [46].

Though competition and environmental filtering are often thought of as dominant forces in the structuring of communities, there are other factors that could come into play in this system and others. Predation, or herbivory in this case, could be involved in the structuring of communities. Herbivory could promote phylogenetic overdispersion if herbivores fed on more than one species, and those species tended to be closely related, through the action of ‘apparent competition’ [47], in a manner analogous to resource competition. Theory has shown that apparent competition can act very similarly to resource competition [48], and so limiting similarity may act here as well, only in this case the similarity is in shared predators rather than shared resources. This effect can also be thought of as a phylogenetic extension of the Janzen-Connell hypothesis [49], [50] as described in a recent review of community phylogenetics [8]. Such “Janzen-Connell” effects could be stronger in the undisturbed plots. Though it is unlikely that herbivore pressure differs greatly between the habitats (given their close spatial proximity), herbivore effects would likely be gradual and only result in significant difference in community structure over many years – years which the undisturbed plots have experienced and which recently disturbed plots have not.

Predation could also act as a filter. Though smaller specialized herbivores such as insects may not vary between the habitats, deer herbivory may. Deer are common on the property on which I conducted my surveys and they may impose pressure on old field plant communities. The increased exposure of disturbed fields makes plants more apparent, and so deer could be a stronger force in recently disturbed plots. If traits that lead to deer-resistance are phylogenetically conserved, deer could act as a filter leading to phylogenetic clustering in areas where deer are more common.

Many of the effects discussed above vary in the timescale over which they act. Most of the filters promoting clustering will act immediately, whereas those thought to promote overdispersion will act gradually. It may be useful to define filters as density-independent effects on fitness, whereas competitive effects (including both resource and ‘apparent’) are density-dependant, in that they become stronger in high densities. It may be then that disturbance exposes plant communities to environmental filters which leads to greater than expected phylogenetic clustering at low densities, after which communities gradually return to their ‘expected’ level of phylogenetic structure, through the action of weak dispersion promoting forces, such as limiting similarity and Janzen-Connell effects, which become important as densities increase. It is particularly interesting to note however, that in this system this process leads to no difference in the absolute phylogenetic diversity of the different habitats, due to differences in their species pool and therefore differences in the expected phylogenetic structure of each habitat.

This could have implications for conservation. It is becoming clear that phylogenetic diversity has consequences for ecosystem functioning [51], [52]. If so, reductions in phylogenetic diversity could have negative effects that may be independent of the effects of species richness. Indeed, one study found that urban areas (assumed to be more disturbed) actually had higher species diversity of plants but that phylogenetic diversity was lower [21]. This likely reduces the positive aspects of increased species richness. Consistent with many other studies [17]–[19], [21], I demonstrated the ability of disturbance to decrease the phylogenetic diversity of an area, however, I also show that whether this leads to an absolute difference in phylogenetic diversity between disturbed and undisturbed habitats is dependant on the pool of available species in each habitat. In this case, disturbed areas had the potential for higher phylogenetic diversity than undisturbed areas, but clustering at the plot level led to statistically indistinguishable values for phylogenetic diversity in each habitat.

### Conclusion

Factors that influence ecological succession may act in a biased manner with respect to phylogeny, because of a correspondence between phylogeny and ecological similarity. Therefore, phylogenetic information should be useful in understanding these forces. In this study I found phylogenetic information could be used to get a fuller picture of compositional changes in plant communities. In particular, disturbed plant communities were more phylogenetically clustered than expected by chance, suggesting the action of environmental filters on phylogenetically conserved traits. Importantly, this led to no difference in phylogenetic diversity between disturbed and undisturbed plots, because the underlying species pool for disturbed plots had a higher phylogenetic diversity. This suggests that processes that structure communities can have different effects on phylogenetic diversity at different scales, from the regional to the plot level. This necessitates the careful choice of null models when comparing phylogenetic diversity indices amongst habitats. Analyzing differences in phylogenetic structure and composition at different scales can lead to useful insights into habitat differences in community composition.

## Supporting Information

Data S1 Raw Data. Raw data used in the study (comma delimited text file). Columns are species denoted by their binomial latin name. Rows are the plots sampled. Value in each cell is the number of quadrants in the plot the species was found in (0–4).
(0.01 MB CSV)

Data S2 Phylogeny. Phylogeny file used in this study (in Newick format). Note: Thuja occidentalis not included.
(0.00 MB TXT)
